# Groundtruth: A Matlab GUI for Artifact and Feature Identification in Physiological Signals

**DOI:** 10.3389/fphys.2019.00850

**Published:** 2019-08-20

**Authors:** Ganesh R. Naik, Gaetano D. Gargiulo, Jorge M. Serrador, Paul P. Breen

**Affiliations:** ^1^Biomedical Engineering and Neuromorphic Systems, The MARCS Institute for Brain, Behaviour and Development, Western Sydney University, Penrith, NSW, Australia; ^2^Rutgers Biomedical and Health Sciences, Newark, NJ, United States; ^3^Department of Pharmacology, Physiology & Neuroscience, New Jersey Medical School, Rutgers University, Newark, NJ, United States; ^4^Translational Health Research Institute, Western Sydney University, Penrith, NSW, Australia

**Keywords:** graphical user interface, artifacts, physiological signals, Matlab, feature identification, respiratory rates, Bland–Altman plots

## Abstract

Groundtruth is a Matlab Graphical User Interface (GUI) developed for the identification of key features and artifacts within physiological signals. The ultimate aim of this GUI is to provide a simple means of assessing the performance of new sensors. Secondary, to this is providing a means of providing marked data, enabling assessment of automated artifact rejection and feature identification algorithms. With the emergence of new wearable sensor technologies, there is an unmet need for convenient assessment of device performance, and a faster means of assessing new algorithms. The proposed GUI allows interactive marking of artifact regions as well as simultaneous interactive identification of key features, e.g., respiration peaks in respiration signals, R-peaks in Electrocardiography signals, etc. In this paper, we present the base structure of the system, together with an example of its use for two simultaneously worn respiration sensors. The respiration rates are computed for both original as well as artifact removed data and validated using Bland–Altman plots. The respiration rates computed based on the proposed GUI (after artifact removal process) demonstrated consistent results for two respiration sensors after artifact removal process. Groundtruth is customizable, and alternative processing modules are easy to add/remove. Groundtruth is intended for open-source use.

## Introduction

The recent advances and success of low-cost portable and wearable devices have opened the door to a new generation of medical applications using physiological sensors for health monitoring and biomedical applications. However, these recordings are contaminated by many noise sources, thereby degrading the signals of interest compromising the interpretation of the underlying physiological information (Han and Kim, 2012; Pham et al., 2017; Chavez et al., 2018).

Physiological signals are contaminated by artifacts arising from a variety of sources. These include: (i) movements of the head or body; (ii) bioelectric potentials arising from within the body, e.g., electrical potentials generated by muscles; and (iii) skin resistance changes, e.g., due to perspiration (Talsma and Woldorff, 2005; Sweeney et al., 2012; Noury et al., 2016; Zou et al., 2016; Romano et al., 2018).

Physiological signal trends, dynamics, and correlations reflect the complexity of the underlying physiology (Nizami et al., 2013). Usually, these signals are small in amplitude, thus artifacts arising from environmental, experimental, and physiological factors, can degrade the signal-to-noise ratio and render the affected part of the signal useless. Physiological artifacts can easily interfere with a neurological signal of interest up to the point that they can be mistaken for the source signal, e.g., in the development of Brain-Computer Interfaces (Fatourechi et al., 2007). Hence, the development of algorithms capable of identifying and removing artifacts in live or recorded data is paramount (De Rossi et al., 2003; Zheng et al., 2014). It is critical following appropriate processing to remove noise and artifacts, that features of interest (e.g., R-peaks) are correctly extracted such that diagnosis and/or treatment is provided based on an accurate assessment of the patient. To summarize, it is important to confirm that any automated algorithm developed accurately distinguishes signal artifacts and identifies key physiological features.

This paper presents Groundtruth, a graphical user interface (GUI) for Matlab. Groundtruth has been developed to facilitate signal inspection and identification of artifacts and features in physiological recordings. It allows interactive marking of artifact regions as well as simultaneous interactive removal/addition of marked physiological features, e.g., respiration peaks in respiration signals, R-peaks in ECG signals, etc. Live interactive data processing and display enables faster data processing. The current version includes statistical analysis based on Bland–Altman plots (Connelly, 2008). The system is totally customizable beyond the provided functionalities, and it is possible for the user to implement custom feature identification methods and define new plots to be automatically generated.

The primary purpose of this software is to provide a simple means of determining if a new sensor is theoretically capable of providing an accurate record of a physiological signal, as compared to some other standard method. A by-product of this manual effort is the ground truth of the underlying signal, which can be used as an accurately marked dataset for future comparison with automated algorithms for feature and artifact identification.

## Methods: Basic Program Description

Groundtruth is entirely written in Matlab programming language version 2017b (The MathWorks Inc., MA, United States) on Microsoft Windows 10 (Microsoft Inc., WA, United States). All functions are contained within two m-files, and the complete software package includes GUI-related information and sample data stored in subfolders. However, as the software is intended to be a useful tool for researchers with no specific programming skills, all currently implemented functionalities are directly available from the intuitive GUI.

The GUI permits interactive marking of artifact regions and the detected unwanted features (in this case peaks) and the regions are removed using inspection methods. The processed individual subject results are automatically saved in dedicated folders (or data directory) for further (data or statistical) analysis.

### Graphical User Interface (GUI) Description

**FIGURE 1:**
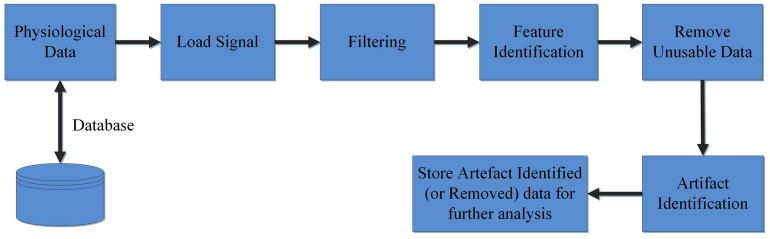
Block diagram of a system process pipeline.

The *Groundtruth GUI* is designed to display multiple channels of physiological signal recordings and allow the user to easily browse their data. Data may be filtered using a built-in console, and the user can display both raw and filtered data. A subsequent console window allows simultaneous artifact and feature identification, as shown in Figure 1. These steps define the basic design and process pipeline of our software package.

#### Loading Raw Data and Filtering

**FIGURE 2:**
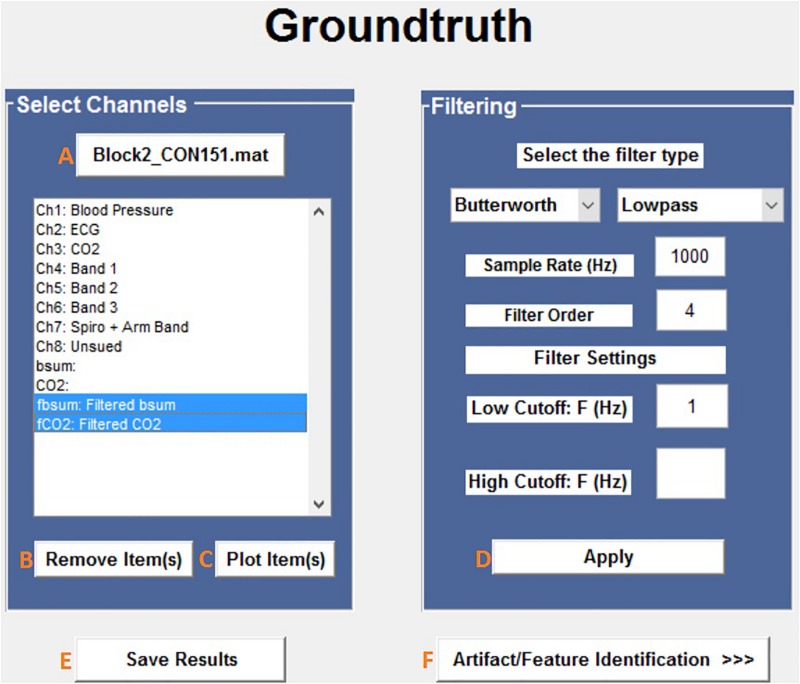
Groundtruth GUI launch window. See Table 1 for function descriptors.

The first steps in the analysis process are to load and filter the physiological data (Figure 2). When new raw data is loaded, a similarly named folder is automatically created in the Matlab working directory. A list box shows all data channels available (Figure 2A), and the user can plot any selected channel, or multiple channels, for immediate inspection (Figure 2C). Unwanted channels can be deleted using the “Remove Items” pushbutton (Figure 2B). Any channel(s) can be selected and Bandpass, Highpass, Lowpass, or Notch filtered. The “Save Results” pushbutton (Figure 2E) allows the user to store the original raw data and filtered results in a separate folder, which is used for further analysis.

**TABLE 1:** Tools and functions available via the Groundtruth launch window.

**Label**	**Button name**	**Description**
A	Load data	Allows the user to load raw data from the directory/folder.
B	Remove item(s)	Allows the user to exclude unwanted channels from the loaded data (list box).
C	Plot item(s)	Allows the user to plot selected channel(s).
D	Apply	Applies the defined filter to the selected individual/multiple channel(s).
E	Save results	Allows the user to save both the original and filtered results in the specified folder. Upon saving, a folder in the name of the data, “*Block2_CON151_ProcData.mat*” (contains the entire channel and filtered data information) and “*Block2_CON151_Results.mat*” (contains only the filtered data results) are created and saved automatically.
F	Artifact/feature identification>>>	Opens a new window for further data analysis.

All data used for artifact/feature identification are stored in.*mat* format. The output (filtered and artifact removed) files are also stored in the same format. All data loaded by Groundtruth are assumed to be column vectors with samples in rows.

#### Loading Raw Data and Filtering

**FIGURE 3:**
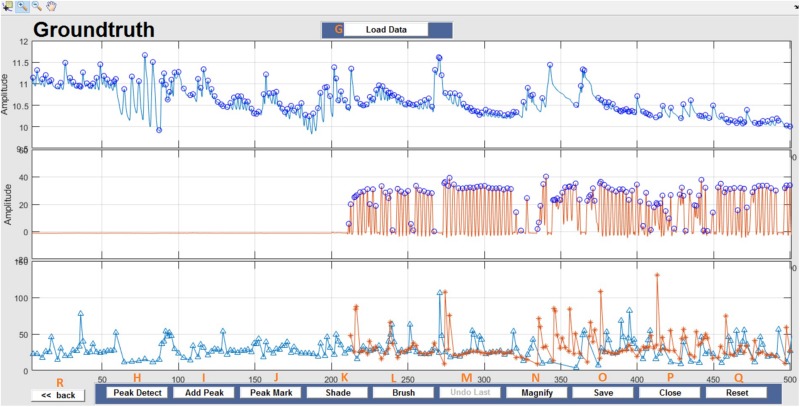
Groundtruth GUI artifact and feature identification window. Top two panels are data selected for analysis in the launch window. The bottom panel shows instantaneous peak frequency which is useful to identify artifacts and misidentified peaks. See Table 2 for function descriptors.

This window enables interactive identification of artifacts and features in physiological signal recordings. The primary functions available within this window are shown in Figure 3 and are briefly explained in Table 2. Physiological feature identification is a time-consuming task, to aid the user automated peak detection may be applied to speed the process (Figure 3H). In the event that a user wishes to inspect or amend previously processed files, previously saved artifacts/features will be marked from stored data (Figure 3G). Unwanted peaks not associated with a clear feature of interest can be removed using “Peak Mark” (Figure 3J). A new peak can be added using the add peak function (Figure 3I). The location of the removed peaks is still retained in a separate.*mat* file. The user can remove unwanted blocks of data using the “Brush” (Figure 3L) function. The most likely scenario for brushing data is in the event that two or more sensors are being compared, but the sensors were not activated simultaneously. In this event, a user may wish to delete (i.e., brush) all data that is not concurrently valid on all channels. Shading is used for marking artifact regions, which are then ignored for further data analysis (Figure 3K). The data is retained such that it may be used for the development of automated artifact rejection. The user can magnify any section of data using “Magnify” (Figure 3N). Once the artifact and feature identification tasks are completed, users can save the results using “Save” (Figure 3O), and the final processed data are stored in the similarly named folder where the initial filter results are stored. In the current GUI, a third axis is introduced to visualize the respiration rate computed for two-axis data. The addition of the axis helps in identifying and removing unwanted features (peaks) rapidly and thus helps in obtaining correct respiration rate results. An illustration of instantaneous respiratory rate detection is shown in Figure 6. In general, very high instantaneous respiration rate points can affect the calculation of respiration rate computed for every minute, thus affect the overall respiration rate (accurate average respiration rate) results. As indicated in the figure, very high instantaneous respiratory rate peaks (mainly due to artifacts) can be removed rapidly to obtain correct respiration rate results.

**TABLE 2:** Tools and features available in the Groundtruth analysis window.

**Artifact analysis GUI functionalities**		
**Label**	**Button name**	**Description**
G	Load data	The user can load two channels of data. In the event that a user wishes to inspect or amend previously processed files, previously saved artifacts/features will be marked from stored data.
H	Peak detect	Runs peak-detect algorithm for the loaded two-channel data.
I	Add peak	Allows the user to add new features (peaks) to two-channel data.
J	Peak mark	Allows the user to mark and remove the unwanted features (peaks) from two-channel data.
K	Shade	Allows the user to shade artifact regions. The shaded regions are ignored in subsequent data analysis.
L	Brush	Allows the user to remove unusable data. The removed regions are either replaced with a user-set constant value or NAN information.
M	Undo last	Undo the most recent brush option performed.
N	Magnify	Magnify a small portion of data.
O	Save	Saves all data, including shaded, brushed, peak marked information in.*mat* format. Data is stored within the same directory as the raw/filtered data.
P	Close	Closes the GUI.
Q	Reset	Reset the signals to their original view.
R	<<Back	Allows the user to return to the *Groundtruth* GUI launch window.

## Example Use Cases

### Respiration Data Analysis – Concussion Study

**FIGURE 4:**
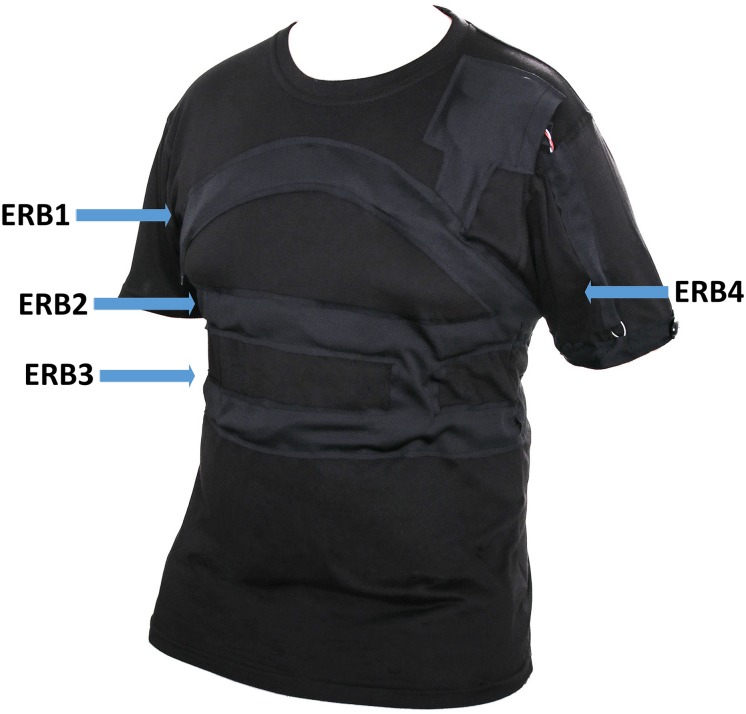
Electro Resistive Band (ERB) embedded T-shirt (image courtesy of Medical Monitoring Solutions Pty Ltd.).

In this section, an illustrative example of artifact and feature identification for physiological data with our software package is presented. The study uses data collected from concussed amateur rugby players using wearable, non-invasive, non-contact and unobtrusive electroresistive polymer sensors embedded in a standard T-shirt (Gargiulo et al., 2015), as seen in Figure 4. Three of the bands were arranged such that the topmost band is under the armpits, while the bottom band is just below the diaphragm. A fourth band, worn on the arm, is unused in this example. This arrangement was chosen because it covers almost the entire volume occupied by the lungs inside the thoracic cage (Gargiulo et al., 2015). The data were recorded from the rugby players following their exit from the game. During the experiments, the players wore the T-shirt, and the respiratory-related information was recorded with a sampling rate of 1 kHz. As expected during the data recording, artifacts are generated either due to sensor movement or subject body movements. The sum of the data from the three thorax bands provides information about respiration and are compared with nasal CO_2_ data. The utility of the proposed Matlab GUI for artifact and feature identification is demonstrated in this simple example as a means of validating the ability of the t-shirt to accurately capture respiratory rate in six subjects. Ethical approval was obtained from the Rutgers University Institutional Review Board and the Western Sydney University Human Research Ethics Committee, and all subjects provided written informed consent.

**FIGURE 5:**
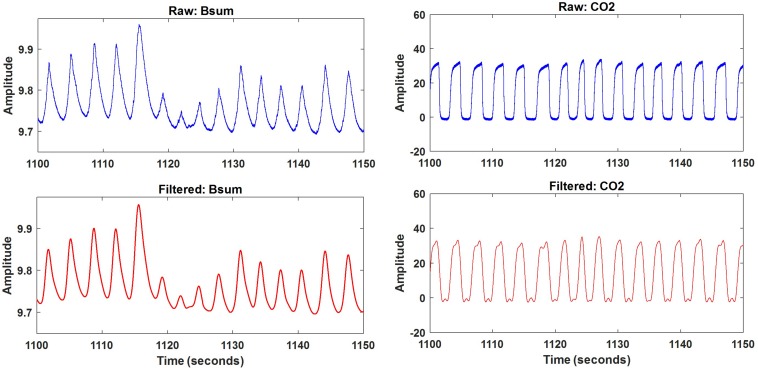
An example of the raw and filtered sum of bands data (left) and CO_2_ (right).

Once the data file was opened, the samples to be analyzed (sum of bands Bsum and nasal CO_2_) were automatically selected. Both Bsum and CO_2_ data were filtered using a lowpass filter with a cut-off frequency of 1 Hz. A 50-s excerpt of original and filtered results for one subject is depicted in Figure 5. The filtered signals were saved and further processed using the artifact and feature identification analysis window.

**FIGURE 6:**
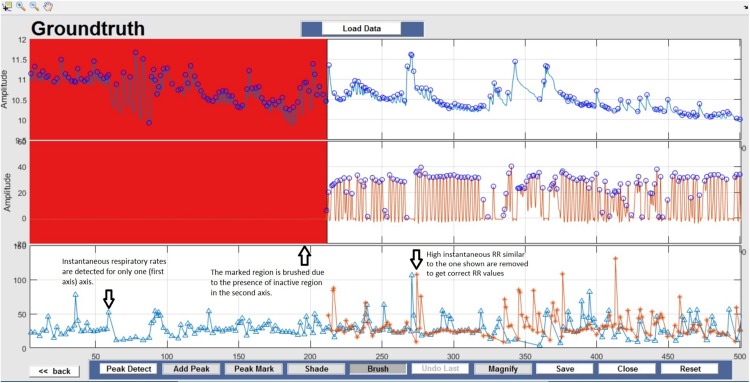
Brush – Unusable data regions are completely removed using Brush and highlighted in red. This action can be reversed by selecting the Undo Last option.

**FIGURE 7:**
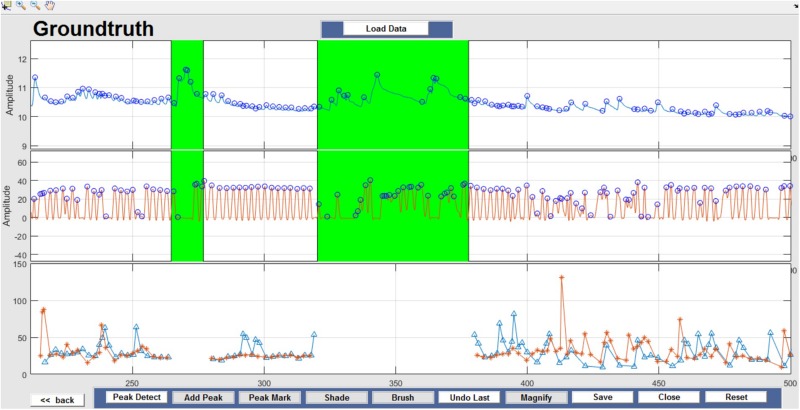
Shade – Data artifacts are marked using Shade and are highlighted in green. Although present in the figure, shaded portions are not considered for further data analysis.

**FIGURE 8:**
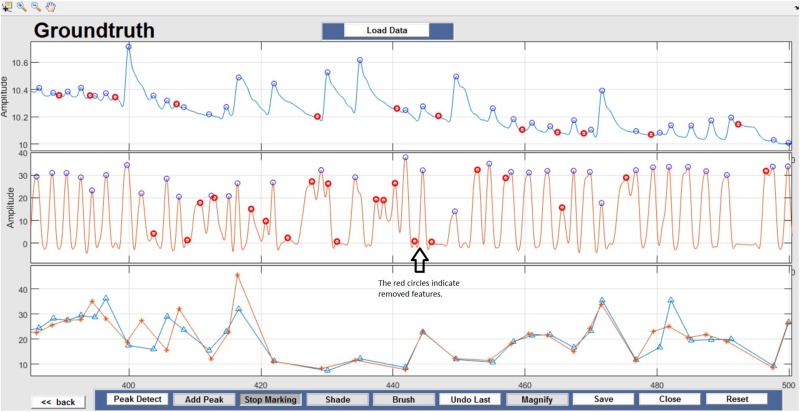
Feature Identification – Circles indicate peaks automatically detected by the Peak Detect algorithm. Incorrectly detected peaks are identified manually by the user and highlighted in red. This action can be reversed by selecting the marked circle again.

**FIGURE 9:**
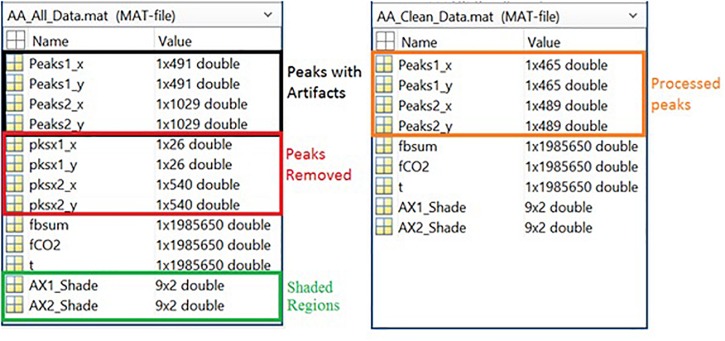
Left Panel: All data information, including peak data, marker data, filtered raw data, and shade locations. Right Panel: Cleaned data information, including peaks and filtered raw data.

Respiration peaks were initially detected using the inbuilt window-based threshold method (Peak Detect). To clean the data and identify the ground truth, data analysis was carried out in three steps. First, in the initial data segments of data (0–210 s in Figure 6), it is clear that the CO_2_ sensor was not connected (middle panel). As there is no useful comparison that can be made here, and it is not an artifact, we delete this data completely using Brush (Figure 6). Second, regions with clear artifacts are identified and marked using Shade (Figure 7). Third, feature identification is performed by removing peaks/incorrect features (Figure 8). In this example, it is clear that the automated peak detection identifies many more features in the data than those of interest (i.e., respiration cycles). The processed data are stored in the same directory as the original data along with the final output figure. The processed data are stored in two Matlab files in the.*mat* format (see Figure 9), “*AA_All_data.mat*” containing both the raw and clean data and “*AA_Clean_data.mat*” containing only clean data. Figure 9 (left) shows the information about peaks with artifacts, peaks removed, and shaded regions, whereas Figure 9 (right) depicts the cleaned signal. AX1_Shade and AX2_Shade are arrays indicating the start/stop times of marked artifact (shaded) regions.

Instantaneous breath-by-breath respiratory rates are subsequently obtained from the position of two consecutive respiratory peaks (*P*_*i*_ and *P*_*j*_) and the sampling rate (*F*_*S*_ = 1000 Hz). The time difference Td between the two peaks can be calculated using the following formula:

(1) $$T_{d}=\frac{\left|P_{i}-P_{j}\right|}{F_{s}}$$

The instantaneous respiration rate (*F*_*R*_) from raw (but filtered) data is then calculated by taking the inverse of *T*_d_ i.e., *F_*R*_* = *1/T*_d_. The respiration rate is then calculated for 60s intervals (breaths/min) and averaged (multiple 60 s intervals) over the entire length of data. The impact of data processing can then be compared as can the performance of the two sensor types.

### ECG Data Analysis – Physionet Database

The identification of a representative ECG signal may be affected or even compromised by the presence of noise and artifacts. The sources of these artifacts can be physiological, such as muscle activity or skin movements, or non-physiological as a result of neighboring electrical devices or incorrect use of the equipment. Traditional signal processing methods such as a notch, DC and bandpass filtering help in eliminating these artifacts mentioned to a certain extent, but for clinical diagnosis, it would be ideal to obtain clear ECG signal, and following this reasoning, we feel Groundtruth GUI can help clinicians to achieve that task.

**FIGURE 10:**
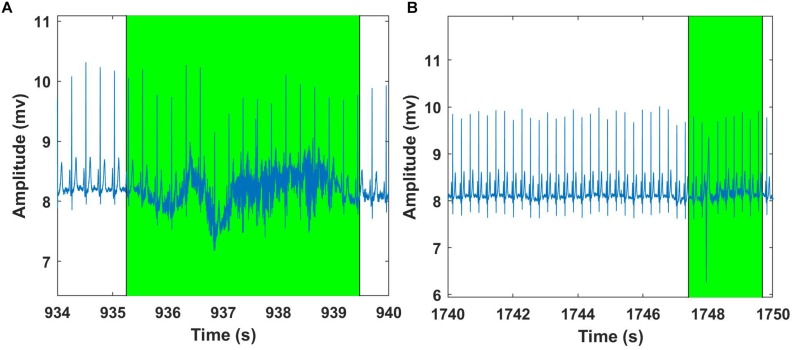
Examples of Fantasia – ECG signal analysis using Groundtruth GUI: An excerpt of ECG artifact shaded region – young subject **(A)**. Another example showing ECG artifact shaded region – older subject **(B)**. ECG artifacts are marked using Shade and are highlighted in green. The shaded regions are discarded for the HR calculation.

In this section, artifact and feature identification (heart rate) for ECG data using our Matlab GUI are presented. We have used the Fantasia database (available for free from the Physionet database) for ECG analysis (Goldberger et al., 2000). The database is composed of 40 ECG waveforms recorded for 2 h from healthy volunteers during supine resting while watching the movie Fantasia (Disney et al., 1940). This dataset was divided into two groups: young (21–34 years old) and elderly (68–85 years old). For simplicity, we chose a dataset consisting of 20 subjects (10 young and 10 old). Each group contains the same number of men and women. All subjects were in a supine position while watching the movie, and the signals were recorded for about 2 h with a sampling frequency of 250 Hz. The beats from each ECG were carefully cataloged through unsupervised systems followed by visual inspection of experts. The fantasia database consists of simultaneous recordings of ECG and respiration data. However, we aimed to test the efficacy of the ECG signal using our GUI and hence, only ECG data were considered for further analysis. Before Groundtruth analysis, the raw ECG data was filtered using notch (50–60 Hz) and DC filters to remove power line noise and baseline wandering. The filtered ECG signals were saved and further processed using the artifact and feature identification analysis window. The regions with clear artifacts are identified and marked using Shade Before Groundtruth analysis, the raw ECG data was filtered using notch (50–60 Hz) and DC filters to remove power line noise and baseline wandering (Figure 10); the processed ECG data are stored in the same directory as the original data. The HR was computed for processed (before and after artifact) ECG data using modified Pan-Tomkins algorithm for every minute (beats/minute) and averaged (multiple 60 s intervals) over the entire length of data (Pan and Tompkins, 1985).

## Results

### Respiratory Rate

The mean respiratory rate for the six concussed rugby players calculated from the raw (filtered) and fully processed data are reported in Table 3. Data is shown for calculations based on the CO_2_ sensor and shirt data.

**TABLE 3:** Mean respiratory rates.

**Subjects**	**Mean respiratory rate (breaths/min)**			
	**Raw data**		**Processed data**	
	**Bsum**	**CO_2_**	**Bsum**	**CO_2_**
1	22.51	42.86	11.93	12.73
2	19.03	43.83	14.55	14.76
3	21.39	42.23	15.79	15.86
4	30.01	43.80	20.56	20.63
5	37.39	38.00	19.46	19.62
6	30.82	33.00	20.38	20.76
Mean ± SD	26.86 ± 7.02	40.62 ± 4.31	17.11 ± 3.56	17.39 ± 3.4

### Heart Rate

The mean heart rate for the twenty (10 young and 10 old) subjects calculated from the raw (filtered) and fully processed data are reported in Table 4. Data is shown for calculations based on the ECG data. From the results (Table 4) it can be seen that HR values have been slightly increased after the artifact removal (ignoring the shaded regions), this is due to the fact that during standard HR analysis some of the QRS regions were missed due to huge artifact regions and thus missed some QRS peaks.

**TABLE 4:** Mean heart rates.

**Subjects**	**Mean heart rate (beats/min)**			
	**Young subjects**		**Old subjects**	
	**HR - before groundtruth GUI**	**HR - after groundtruth GUI**	**HR - before groundtruth GUI**	**HR - after groundtruth GUI**
1	72.32	73.23	59.34	62.36
2	58.35	60.45	56.51	60.01
3	63.05	65.36	59.79	60.89
4	45.71	48.34	51.69	53.32
5	57.39	60.27	55.23	58.31
6	58.93	61.23	51.43	53.26
7	49.35	52.34	59.33	61.45
8	59.98	62.23	70.69	71.45
9	66.68	68.76	40.83	44.65
10	72.02	72.94	68.17	69.23
Mean ± SD	60.38 ± 8.67	62.52 ± 8.07	57.30 ± 8.52	59.49 ± 7.82

### Bias and Agreement

The Bland–Altman method was used to determine bias (mean difference between methods) and limits of agreement (±95% confidence around bias) for comparison of respiration rates from original and processed data. This method provides a quantitative and visual comparison of two measurements by plotting the differences between the two techniques against the average of the two methods. It supplements correlational analyses by determining the limits of agreement between methods (in which the Mean level ±1.96 SD represents bias ±95% confidence interval range of agreement).

**FIGURE 11:**
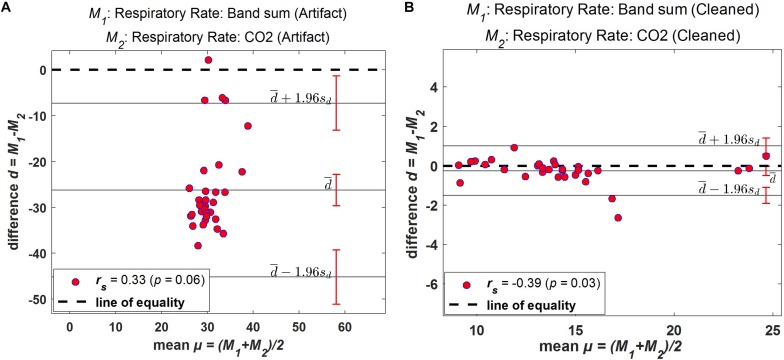
Bland–Altman plots. Difference between windowed Bsum and CO_2_ breaths/min plotted against the average value of the two methods for one subject. **(A)** Original data (filtered but otherwise unprocessed); **(B)** Fully processed data with artifacts marked and ignored. Solid horizontal lines indicate mean difference and upper/lower limits of agreement, sd: standard deviation.

Bland–Altman plots demonstrate the extent of bias and agreement between original and processed results for mean respiration rate calculated in 60-s windows. The result for one subject is depicted in Figure 11, where the mean difference between the Bsum and CO_2_ for original data (filtered but otherwise unprocessed) was 26.22 breaths/min (95% agreement limit −45.19–7.272; Figure 11A). In contrast, the analysis showed that the mean difference between the Bsum and the CO_2_ for the cleaned data was far narrower (mean −0.25; 95% agreement limit −1.51–1.01; Figure 11B).

**FIGURE 12:**
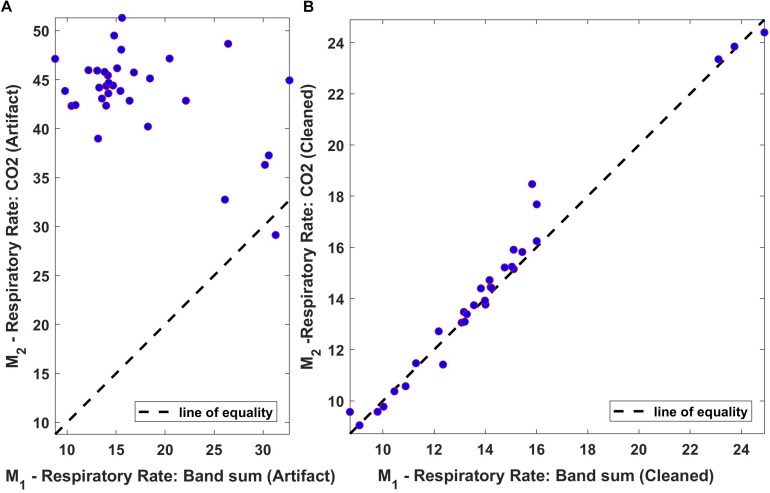
Regression plot of the mean respiration rate of Bsum and CO_2_ (breaths/min). **(A)** Original data (filtered but otherwise unprocessed); **(B)** Fully processed data with artifacts marked and ignored. Dotted lines indicate the regression line.

The relationship between averaged values of “Bsum” and “CO_2_,” computed over 60-s intervals, was also assessed using a linear regression scatter plot. The scatter plot of the respiration rate for both original and processed data from one of the subjects is shown in Figure 12. Ideally, the two techniques would exhibit perfect agreement, and when the data is plotted as a scatter plot, all points would lie on the line of equality. From the results, it is clear that the two methods are in good agreement following processing.

## Discussion

The Groundtruth toolbox provides a range of Matlab functions enabling convenient artifact and feature identification in a broad range of physiological data. Together with the brief theoretical and practical context provided in this paper, it is well placed to facilitate efficient progress in a wide cross-section of the physiological science and engineering communities. However, it is critical to ensure that the toolbox is not treated as “a black box” whereby its output is assumed to be a valid and informative transformation of its input. As with any complex physiological signal analysis, the toolbox incorporates a comprehensive range of validation and pre-processing tools. Hence, depending on the type of physiological signal, users can modify the toolbox accordingly to fit their requirements and applications.

Precise and reliable artifact/feature identification is a significant requirement for automated and intelligent physiological signal monitoring. No alternative method exists to establish an empirical dataset based on recorded data. Expert annotated data is available for select data, e.g., EEG data via Physionet, which may be used in the development of new software algorithms. However, developers of new sensors, amplifiers, or other hardware have no such luxury and must find a means of accurately annotating their (own) data. Our motivation in developing Groundtruth was to address this problem and provide an efficient means of marking artifacts and features in long-term physiological recordings from new sensor technologies. The flexibility to create custom analytics, specific to the requirements of the system under test, within a structured data environment is highly valuable in the development of new physiological sensors. Furthermore, a dataset of key features/artifacts reduces workload considerably if/when an alternative analytical approach is pursued, as often the same marked features/artifacts can be reused. Next, we explain how Groundtruth can be modified to handle numerous physiological signals.

### Future Improvements

**FIGURE 13:**
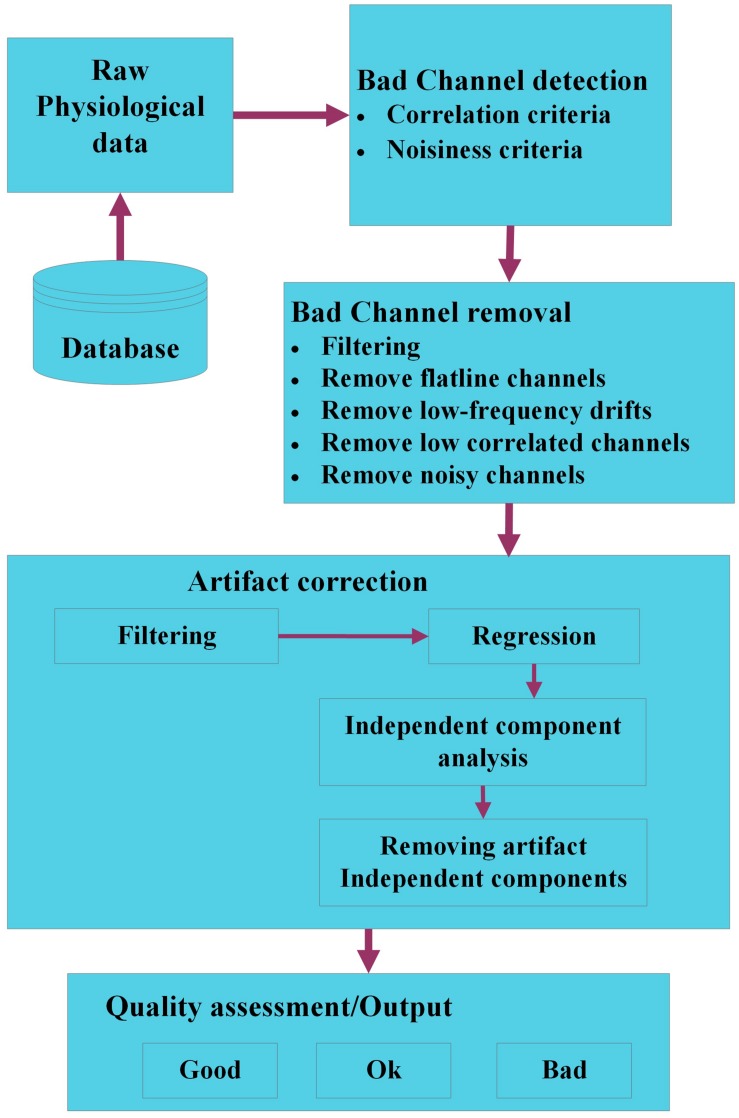
The workflow of a modified Groundtruth GUI (future use). The raw physiological data are organized in a database. Bad channels within each dataset are identified using signal processing and statistical methods; hence, noisy, and outlier channels are removed. The artifact data are corrected using filtering, regression, and blind source separation/independent component analysis methods, and various artifact components are removed. Finally, the quality of each dataset is assessed using objective criteria to categorize data as “good,” “ok,” or “bad” data.

Groundtruth is a novel MATLAB toolbox developed for easy and reliable identification features, artifacts in numerous physiological signals (respiration, ECG, PPG, etc.). The intuitive interface includes features that are absent from widely used contemporary artifact removal softwares, but still essential to researchers due to various simple to use functions. This version of Groundtruth is in infancy and currently work is in progress to extend its functions using advanced signal processing methods such as blind source separation (BSS), independent component analysis (ICA), Wavelets and adaptive filtering methods. An outline of the planned extension of Groundtruth using various signal processing methods is depicted in Figure 13.

Initially, physiological data are loaded from the database; in a first step, “bad channels” are identified. Bad channels are those with low signal to noise ratio (SNR) or part of the signal with no data. If there are only a few channels are affected with low SNR or no data then it is impracticable to remove the entire signal, hence either the signals will be “shaded” or it will be more sensible to interpolate the signal of bad channels by the combined signal of channels with adequate SNR using correlation or standard SNR adjustment criteria. Next, bad channels will be removed using various methods such as filtering, correlation, etc. We will ensure that the bad channel identification procedures only detect bad channels and do not modify (e.g., interpolate channels) of the physiological data. Some of the standard algorithms such as one proposed by Bigdely-Shamlo et al. (2015) will be implemented in the modified GUI. Artifacts are identified using standard/modified BSS or ICA using back projection methods, and affected artifact signals (independent components) are removed either automatically or using threshold techniques. Finally, the quality of each dataset is assessed using objective criteria to categorize data as “good,” “ok,” or “bad” data (Cong et al., 2011; Erhardt et al., 2011).

## Conclusion

In this paper, we presented the Groundtruth GUI, together with an example of its use for two simultaneously worn respiration sensors. The respiration rates are computed for both original as well as artifact removed data and validated using Bland–Altman plots. The respiration parameters computed based on the proposed GUI after artifact removal process demonstrated consistent results for two respiration sensors after artifact removal process as compared to the respiration rate values before the artifact removal.

The current Groundtruth toolbox is in its infancy and is limited in its functionality. It only provides a means for relatively simple artifact and feature identification. However, substantial opportunities exist to extend the functionality of the system. For example, there is scope to incorporate an automated artifact identification process. Methods such as dynamic time warping (Müller, 2007; Li and Clifford, 2012), MOTIF (Federico and Pisanti, 2011; Sivaraks and Ratanamahatana, 2015), change detection (Delorme et al., 2007), etc., could be added as some additional elements in the processing pipeline, with direct functional comparison made at the time of analysis. Performing these analyses remains an active area of investigation. Groundtruth is available free of charge for non-commercial use.

## Data Availability

The datasets generated for this study are available on request to the corresponding author. Some of the supplementary data used in this article named “Supplementary Data Sheet 1” is made available in the website link in Supplementary Material section.

## Ethics Statement

This study was carried out in accordance with the recommendations of the “Rutgers University Institutional Review Board and Western Sydney University Human Research Ethics Committee” with written informed consent from all subjects. All subjects gave written informed consent in accordance with the Declaration of Helsinki. The protocol was approved by the “Rutgers University Institutional Review Board and Western Sydney University Human Research Ethics Committee.”

## Author Contributions

GN carried out the data analysis and wrote the manuscript. PB, GG, and JS guided the analysis. GN, GG, and PB conceived the experiments and edited the manuscript. GG and PB supervised the study.

## Conflict of Interest Statement

The authors declare that the research was conducted in the absence of any commercial or financial relationships that could be construed as a potential conflict of interest.
