# Supplementary figures and images for: Groundtruth: A Matlab GUI for Artifact and Feature Identification in Physiological Signals
Source: Front Physiol. 2019 Aug 20;10:850. doi: 10.3389/fphys.2019.00850 (PMC6710362; doi:10.3389/fphys.2019.00850)

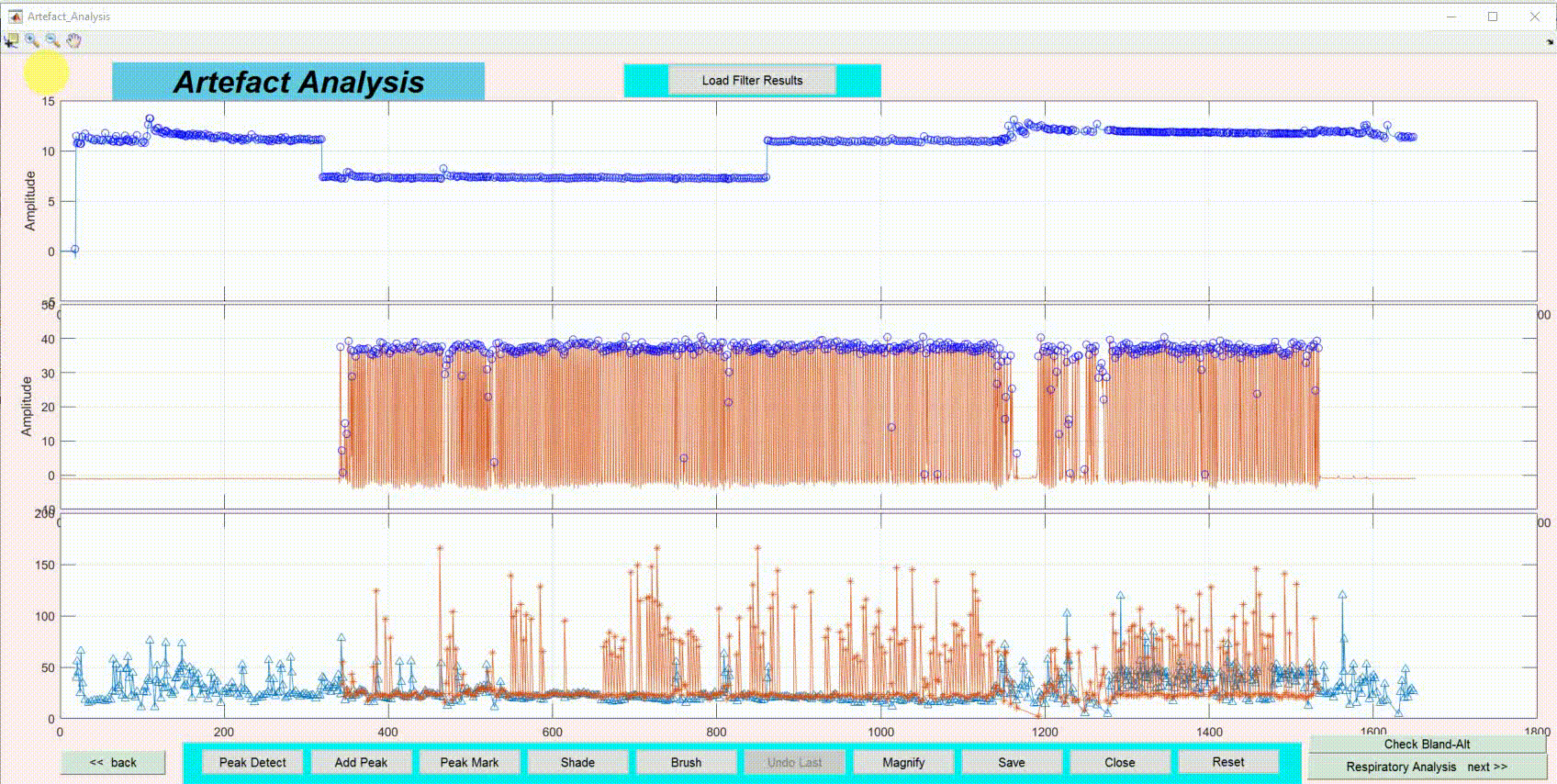

Supplement: Supplementary file 1 [file Data_Sheet_1.ZIP › GUI_Output1.gif]

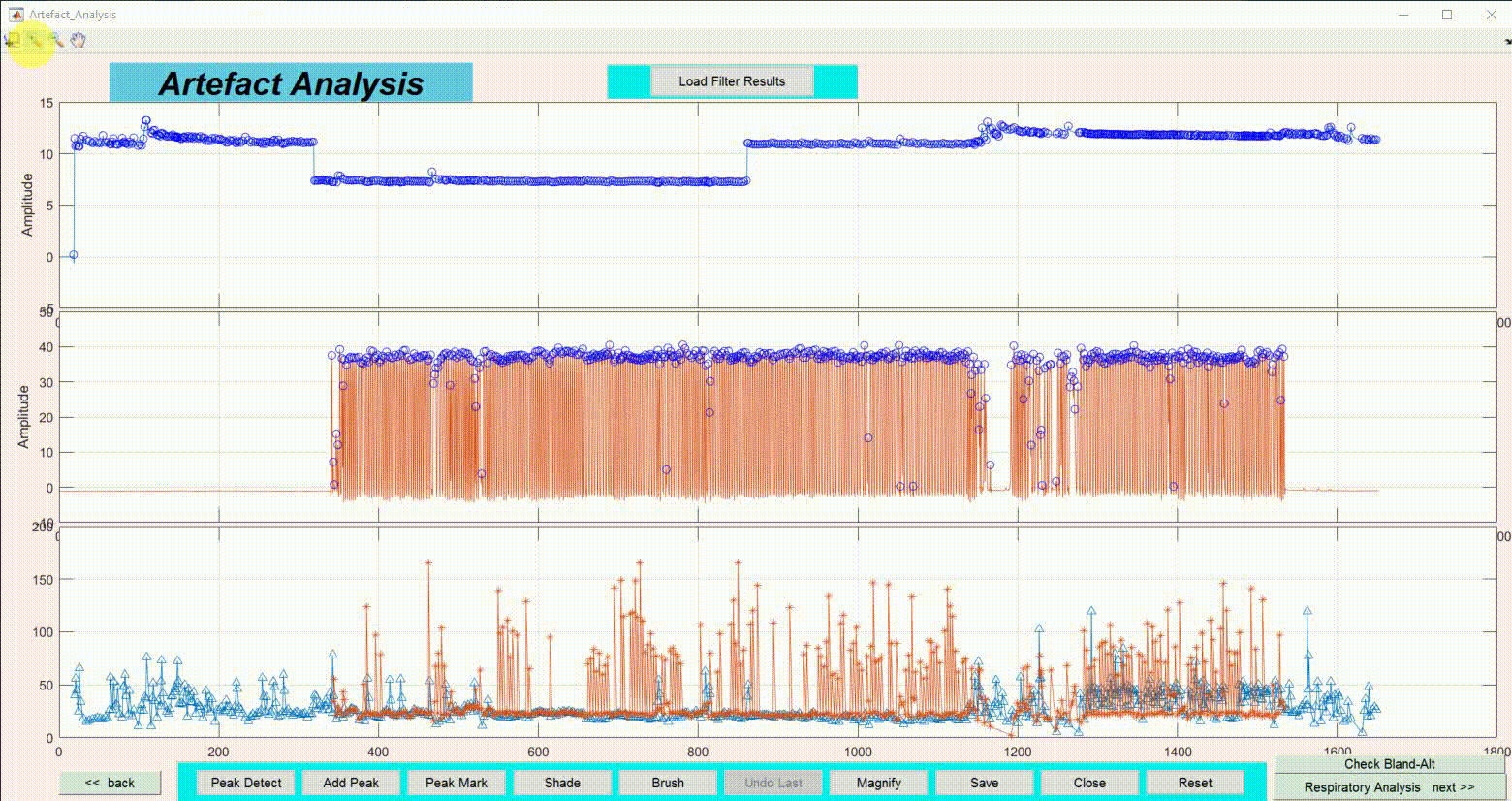

Supplement: Supplementary file 1 [file Data_Sheet_1.ZIP › GUI_Output3.gif]
